# Untying the knot: protein quality control in inherited cardiomyopathies

**DOI:** 10.1007/s00424-018-2194-0

**Published:** 2018-08-14

**Authors:** Larissa M. Dorsch, Maike Schuldt, Dora Knežević, Marit Wiersma, Diederik W. D. Kuster, Jolanda van der Velden, Bianca J. J. M. Brundel

**Affiliations:** Amsterdam UMC, Department of Physiology, Vrije Universiteit Amsterdam, Amsterdam Cardiovascular Sciences, O2 building 11W53, De Boelelaan 1117, 1081HV Amsterdam, The Netherlands

**Keywords:** Cardiomyopathy, Protein quality control, Sarcomeric mutation, Heat shock proteins, Ubiquitin-proteasome system, Autophagy

## Abstract

Mutations in genes encoding sarcomeric proteins are the most important causes of inherited cardiomyopathies, which are a major cause of mortality and morbidity worldwide. Although genetic screening procedures for early disease detection have been improved significantly, treatment to prevent or delay mutation-induced cardiac disease onset is lacking. Recent findings indicate that loss of protein quality control (PQC) is a central factor in the disease pathology leading to derailment of cellular protein homeostasis. Loss of PQC includes impairment of heat shock proteins, the ubiquitin-proteasome system, and autophagy. This may result in accumulation of misfolded and aggregation-prone mutant proteins, loss of sarcomeric and cytoskeletal proteins, and, ultimately, loss of cardiac function. PQC derailment can be a direct effect of the mutation-induced activation, a compensatory mechanism due to mutation-induced cellular dysfunction or a consequence of the simultaneous occurrence of the mutation and a secondary hit. In this review, we discuss recent mechanistic findings on the role of proteostasis derailment in inherited cardiomyopathies, with special focus on sarcomeric gene mutations and possible therapeutic applications.

## Classification of cardiomyopathies

Cardiomyopathies (CM) constitute one of the most common causes of sudden cardiac death in young adults and represent major causes for cardiac transplantation [88]. Disease onset generally ranges between 20 and 50 years of age. CMs are defined by abnormal myocardial structure and function in the absence of any other diseases sufficient to cause these abnormalities [24]. These can be sub-classified based on their functional phenotype and their specific morphological changes. The most common types are hypertrophic CM (HCM), characterized by increased left ventricular (LV) wall thickness often occurring asymmetrically, and dilated CM (DCM), in which the presence of LV dilatation is accompanied by contractile dysfunction [24]. Besides HCM and DCM, there are less frequent forms, such as restrictive CM (RCM) and desmin-related cardiomyopathy [24]. All these cardiomyopathies can be familial and are typically inherited in an autosomal dominant manner. Mutations in genes encoding sarcomeric proteins are the most common cause of these types of CMs [3]. However, the genotype-phenotype relationship is far from clear. The variations in age of CM onset and disease phenotype suggest that additional factors play a role in CM pathogenesis.

Accumulating evidence indicates the presence of derailed proteostasis in CMs as well as its contribution to CM onset and progression. This derailment could either be caused directly by the mutation or indirectly due to a compensatory mechanism. In the former case, the mutant protein might be instable or improperly folded leading to direct activation of the protein quality control (PQC). In the latter case, the mutation does not interfere with protein folding or stability but causes functional impairment, which in turn leads to cellular stress and indirect activation of the PQC. Furthermore, the “secondary-hit” model may apply in CMs, in which a primary sarcomere mutation enhances vulnerability to secondary stressors, which increases cellular burden resulting in PQC derailment. This review summarizes the current knowledge about perturbations in the different components of the PQC in CMs that are caused by mutations in sarcomeric proteins.

## Proteostasis network ensures cardiac health

**Fig. 1:**
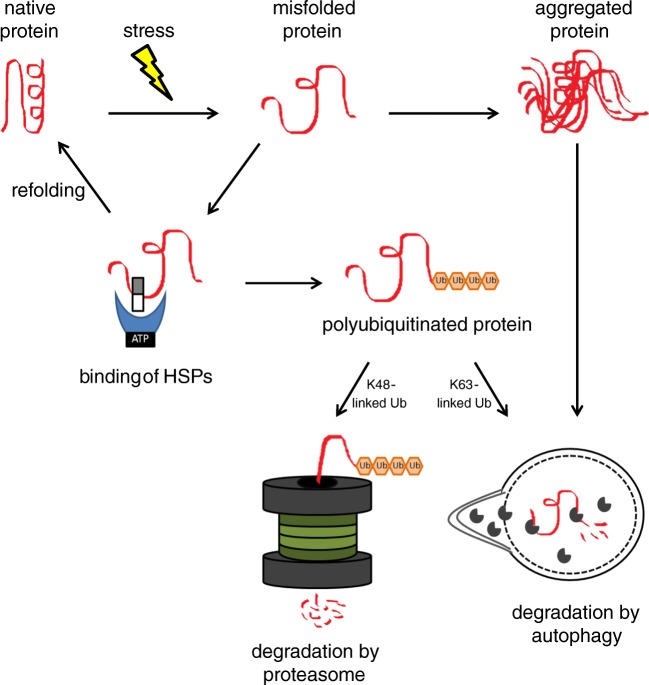
Collaboration of the protein quality control components. Stress leads to misfolding of proteins, which may result in abnormal interaction and subsequent aggregation. Small HSPs (white/gray rectangle) and HSPs with ATPase activity (blue moon shape with black rectangle) prevent aggregation formation by binding to the hydrophobic surfaces of misfolded proteins. They either refold the misfolded proteins to its native structure or initiate its polyubiquitination (Ub, orange hexagon). Misfolded proteins with polyubiquitin chains linked to lysine 48 (K48) are mainly degraded by the proteasome. Misfolded proteins carrying K63-linked polyubiquitin chains and aggregated proteins enter the autophagic pathway

The heart has a very limited regenerative capacity and therefore requires surveillance by a system that maintains protein homeostasis to ensure cardiac health [106]. The PQC system sustains proteostasis by refolding misfolded proteins or removing them if refolding is impossible. It is composed of heat shock proteins (HSPs), the ubiquitin-proteasome system (UPS), and autophagy. PQC is only then functional when all three components are operative and interact with each other. This means that derailment of one of the parts might impair the function of the others in a direct or indirect manner. In a physiological state, protein folding and refolding is ensured by HSPs and their regulators. Terminally misfolded and aggregation-prone proteins are cleared by the two degradation systems, i.e., the UPS and autophagy that work in collaboration with the HSPs (Fig. 1).

First, the different parts of the PQC in normal physiology are described, before addressing their role in CMs.

### Heat shock proteins

HSPs, originally identified as heat responsive proteins, are constitutively expressed in the cell to serve as molecular chaperones to ensure correct folding and assembly of proteins. HSPs are classified in two categories: the small HSPs with a low molecular weight (15–30 kDa) and the chaperones with a high molecular weight (> 30 kDa).

One functional group of HSPs are chaperonin complexes, which are ATP-dependent chaperones with a barrel-like structure that provide correct folding of nascent proteins after translation. Besides their folding function during protein translation, HSPs are also induced in response to cellular and environmental stressors to maintain a healthy cellular proteostasis by clearance of misfolded proteins [66, 111].

**Fig. 2:**
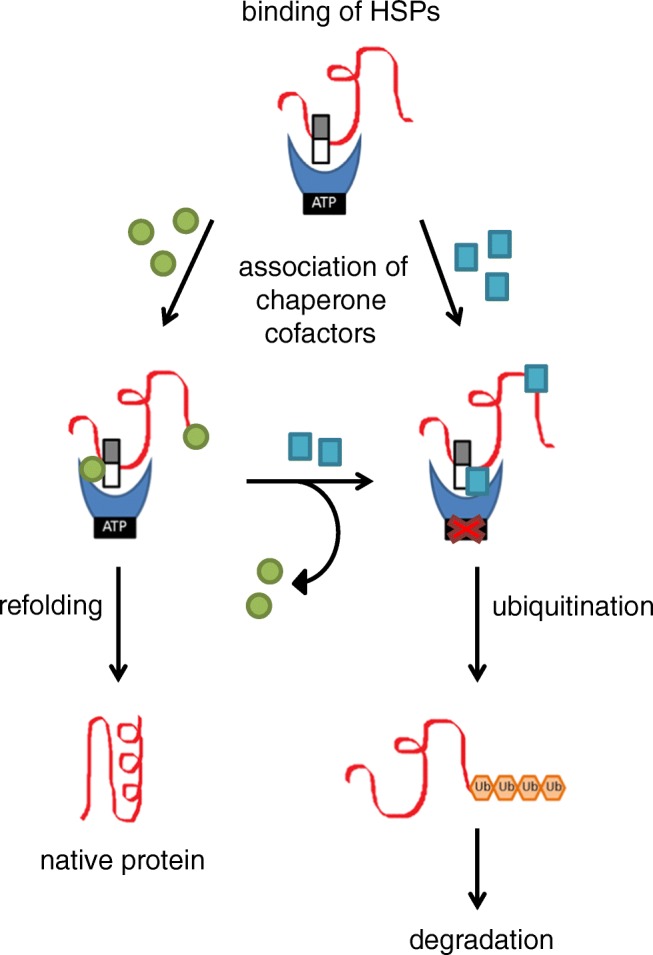
Chaperone cofactor binding determines the heat shock protein (HSP) function. Small HSPs (white/gray rectangle) and HSPs with ATPase activity (blue moon shape with black rectangle) bind to the misfolded protein to stabilize it. Dependent on the chaperone cofactors (green circles or turquoise squares), the misfolded protein gets either refolded or ubiquitinated for subsequent degradation. If refolding is impossible, the chaperone cofactors can be exchanged to promote degradation. In case of ubiquitination, the chaperone cofactors can switch off the HSP refolding activity by blocking the ATPase activity and, together with HSPs, assist in clearance of the misfolded protein via the degradation pathways

As reviewed by Garrido et al., small HSPs show an ATP-independent holdase activity. This means that they bind to misfolded proteins, keep them in a state competent for either refolding or degradation, and thereby prevent or attenuate their aggregation. Due to the association of small HSPs with the HSPs that have an ATP-dependent folding activity, the misfolded proteins can be refolded into their native and functional conformation [28]. The binding affinity of HSPs to the misfolded protein is dependent on the chaperone cofactors bound to the HSPs. Furthermore, this binding of chaperone cofactors determines the processing of the misfolded protein for either refolding or degradation. Chaperone cofactors involved in degradation pathways can switch off the refolding activity of HSPs by inhibiting their ATPase activity and assist the HSPs and the UPS or autophagy in the breakdown of misfolded proteins (Fig. 2) [13, 25]. The degradation of the thick filament protein myosin-binding protein C (MyBP-C), for instance, is mediated via the chaperone cofactor HSC70 playing a major role in regulating MyBP-C protein turnover [32]. These degradation pathways are addressed in the following sections.

To maintain the structure and function of the highly dynamic cardiac sarcomeres, HSPs play an important role. The molecular chaperones GimC (Prefoldin), chaperonin TCP-1 Ring Complex (TRiC), αB-crystallin, and HSP27 ensure correct folding and assembly of proteins, maturation of actin and prevent aggregate formation [10, 14, 34, 36]. HSP27 is mostly found as high-molecular weight oligomers in the cytosol of unstressed cells [23]. Upon stress, HSP27 deoligomerizes and translocates to F-actin and thereby stabilizes the F-actin network [16]. To assemble the myosin thick filament, the chaperones UNC-45, HSP90, and HSP70 are required, whereas the actin filament is self-assembled [7, 8, 94]. Several members of the small HSPs family are expressed in the heart and associate with cytoskeletal proteins [33, 103]. These HSPBs stabilize cytoskeletal structures and improve coping with stress situations [33, 48, 49].

### Ubiquitin-proteasome system

In case of terminally misfolded proteins, that failed be refolded, HSPs and their chaperone cofactors recruit enzymes to mediate polyubiquitination of the target substrate and thereby mark them for the appropriate degradation pathway. Short-lived proteins are typically degraded by the UPS, whereas autophagy is mainly used for degrading long-lived proteins and entire organelles [17, 39].

The polyubiquitination of the target substrate requires the sequential action of three enzymes. The ubiquitin-activating enzyme (E1) activates ubiquitin, which is then transferred to a ubiquitin-conjugating enzyme (E2). In the last step, a ubiquitin ligase (E3) links ubiquitin from the E2 enzyme to a lysine residue of the target protein. There are only two E1 enzymes, several E2 enzymes, and many E3 ligases, each of which recognizes one or several specific protein motifs. Therefore, the substrate specificity is achieved by the selectivity of the different E3 ligases [26, 80]. Dependent on the combination of E2 enzyme and E3 ligase, polyubiquitin chains are linked to the preceding ubiquitin molecule either via lysine 48 (K48) or via lysine 63 (K63), which marks the protein for degradation. Therefore, the polyubiquitination process determines the degradation pathways: Proteins carrying K48-linked polyubiquitin chains are predominantly targeted to proteasomal degradation, and proteins carrying K63-linked polyubiquitin chains enter the autophagic pathways as discussed in the following paragraph [1].

K48-linked polyubiquitinated proteins are transferred to the proteasome, which is almost exclusively the 26S proteasome in eukaryotic cells. This protein complex consists of one 20S core- and two 19S regulatory subunits forming a barrel-like structure. The regulatory subunits have ubiquitin-binding sites to recognize polyubiquitinated proteins and unfold them using their ATPase activity. The unfolded proteins are transferred to the catalytic core and proteolytically cleaved [104].

Sarcomeric proteins have an average turnover rate of 5–10 days [12, 112]. Therefore, they rely on a proper functioning UPS to regulate their clearance. Once dissociated from the myofibrils, ubiquitin-conjugating enzymes mark the proteins for proteasomal degradation by adding K48-linked polyubiquitin chains [91, 112]. In cardiomyocytes, this step is mediated by the MuRF family of E3 ligases [59, 93].

### Autophagy

Autophagy cleans up aggregates or proteins via lysosomal breakdown that cannot be refolded by chaperones or processed by the UPS [46]. During macroautophagy, herein referred to as autophagy, membrane-enclosed vesicles are formed containing the targeted cellular components. First, an isolation membrane is formed engulfing the cytoplasmic material. The membrane expands until the edges fuse to form the autophagosome [118]. Fusion of the autophagosome with a lysosome leads to an autolysosome, which breaks down the cargo [5]. During selective autophagy, proteins carrying a K63-linked polyubiquitin chain are degraded. In the case the proteasome is overwhelmed with proteins carrying a K48-linked ubiquitin chain, such as aggregated proteins, they can also be cleared via autophagy. Their polyubiquitin chain docks to the adaptor protein p62/SQSTM1, which enables the translocation into the autophagosome [45]. Acidic lysosomal hydrolases degrade the captured material together with the inner membrane, and the resulting macromolecules are recycled into ATP, amino acids and fatty acids.

Autophagy is found to be upregulated in response to starvation, growth factor withdrawal, or high bioenergetic demands [50, 56, 65, 86]. The ability to sequester and break down entire organelles, such as mitochondria, peroxisomes, endoplasmic reticulum, and intact intracellular microorganisms, makes autophagy a unique and essential process in the cell. Especially in post-mitotic cells like cardiomyocytes, basal activation of autophagy is important to maintain a balanced proteostasis by degradation of long-lived proteins, lipid droplets, and dysfunctional organelles [17].

Cardiomyocytes have a low basal autophagic activity under normal conditions. Upon stress, the formation of protein aggregates is facilitated and triggers activation of autophagy [96]. Furthermore, cardiac autophagy is initiated in response to energy stress during periods of nutrient deprivation or high metabolic demand [35].

## Proteostasis derailment in inherited cardiomyopathies

The PQC is of great importance in many cardiac diseases caused by “wear and tear,” including cardiac amyloidosis, myocardial infarction, and atrial fibrillation [37, 60, 73, 115]. The activation of PQC in a variety of cardiac stress conditions can be considered as a positive compensatory response to maintain proteostasis. This might be especially true in the case of inherited CMs, where mutant protein expression is the disease-causing mechanism. Recent studies provide evidence for a causative role of the PQC in CM. On one hand, mutations in components of the PQC itself can cause CM. This has been described for the R120G mutation in *CRYAB* encoding for the chaperone αB-crystallin, causing desmin-related CM, and the P209L mutation in the chaperone cofactor *BAG3*, leading to juvenile DCM [87, 105]. Mutations in PQC components as causes of inherited CM are rare, but PQC impairment can also occur as a result of CM-causing sarcomeric mutations. In this case, mutant sarcomeric proteins may impair the function of the PQC through overload of its components including HSPs, UPS, and autophagy. This could lead to increased levels of mutant protein, exacerbating CM disease progression.

So far, the role of PQC has been investigated only in a limited number of studies on CM caused by sarcomeric gene mutations. In vitro information is available for HCM- and DCM-causing mutations in *ACTC1*. Furthermore, it has been studied in vivo with HCM-causing mutations in *MYBPC3*, *MYH7*, and *MYOZ2*; DCM-causing *NEBL* mutations; and RCM-causing *TNNI3* mutations (Table 1). In the following sections, the interaction between CMs and derailments of the different parts of the PQC are described in detail.

**Table 1:** Overview of structural changes and adaptations in the protein quality control system related to cardiomyopathies

Gene	Phenotype	Morphological abnormalities	Chaperones	UPS	Autophagy
*ACTC1*	HCM	not reported	+ (in vitro) [102]	not reported	not reported
	DCM	not reported	+ (in vitro) [102]	not reported	not reported
	RCM	not reported	not reported	not reported	not reported
*ACTN2*	HCM	cytoplasmic vacuolization, perinuclear halo, dysmorphic nuclei (human) [31]	not reported	not reported	not reported
	DCM	not reported	not reported	not reported	not reported
*MYBPC3*	HCM	large irregular vacuoles (infant) [109]	αB-crystallin ↑ (mice) [116]	↓ (mice) [85]	↑ (human) [92] ↓ (mice) [85]
	DCM	not reported	not reported	not reported	not reported
*MYH6*	HCM	not reported	not reported	not reported	not reported
	DCM	not reported	not reported	not reported	not reported
*MYH7*	HCM	not reported	not reported	not reported	↑ (human) [92]
	DCM	not reported	not reported	not reported	not reported
	RCM	not reported	not reported	not reported	not reported
*MYL2*	HCM	not reported	not reported	not reported	not reported
*MYL3*	HCM	not reported	not reported	not reported	not reported
	RCM	ultrastructural defects (mice) [119]	not reported	not reported	not reported
*MYOZ2*	HCM	not reported	not reported	↑ (mice) [41]	not reported
*NEBL*	HCM	myocyte vacuolization (human) [75]	not reported	not reported	not reported
	DCM	enlarged and deformed mitochondria, lipid accumulation (mice) [78]	not reported	not reported	abnormal lysosomes (mice) [78]
*TNNC1*	HCM	not reported	not reported	not reported	not reported
	DCM	no evidence of vacuolization (human) [43]	not reported	not reported	not reported
	RCM	degeneration of myocardial fibers (human) [76]	not reported	not reported	not reported
*TNNI3*	HCM	not reported	not reported	not reported	not reported
	DCM	not reported	not reported	not reported	not reported
	RCM	irregularly shaped megamitochondria (human) [117]	not reported	↓ proteasomal activity (mice) [20]	not reported
*TNNT2*	HCM	myocyte atrophy (mice) [97]	not reported	↓ (mice) [30]	not reported
	DCM	not reported	not reported	not reported	not reported
	RCM	abnormal mitochondria (human)* [74]	not reported	not reported	not reported
*TPM1*	HCM	nuclear gigantism [68]	not reported	not reported	not reported
	DCM	accumulation of TPM1 (mice) [79]	not reported	not reported	not reported
	RCM	not reported	not reported	not reported	not reported

Mixed genotypes are indicated with “*” and a “+” indicates a positive finding

### Diverse abnormalities in heat shock protein function

HSP impairment or activation contribute to disease pathology in CMs. Desmin-related CM displays HSP impairment and is either caused by mutant desmin itself or mutant chaperone αB-crystallin. In a normal state, αB-crystallin binds to desmin and thereby prevents its aggregation [10]. Mutant desmin, however, impairs the interaction with αB-crystallin leading to desmin accumulation and cardiomyocyte dysfunction [54]. This suggests aberrant protein aggregation can cause CM. Correspondingly, the R120G mutation in *CRYAB* results in desmin-related-CM as well and also presents with aggregates containing desmin and mutant αB-crystallin [105]. Sanbe et al. showed that upregulation of HSPB8 due to geranylgeranylacetone treatment reduces the amount of mutant αB-crystallin-containing aggregates [83]. This implies that other HSPs can compensate for the loss of function to remove aggregates. Furthermore, in vitro experiments have shown that HCM- or DCM-causing mutations in *ACTC1*, encoding cardiac actin, can interfere with its folding by the TRiC chaperonin complex resulting in inefficient incorporation of actin into the myofilament and its subsequent aggregation [102]. Mutations in one specific subdomain of actin affect protein stability or polymerization, making actin more prone for degradation. Whereas mutations in other subdomains of actin cause alterations in protein-protein interactions [67]. A gene co-expression analysis of human controls and HCM samples identified the TRiC chaperonin complex as the most differential pathway, thereby further highlighting its importance in HCM [19].

By contrast, various studies on the role of PQC in CMs report on increased levels of HSPs due to PQC activation. However, it still remains unresolved whether the increased levels of HSPs are a direct effect of the mutant protein or a compensatory secondary effect due to increased cellular stress. Therefore, the direct interaction of mutant protein and HSPs needs to be studied. In mice with a truncating *MYBPC3* mutation and an HCM phenotype, increased levels of αB-crystallin have been found [116]. In other CM mouse models, independent of a sarcomeric mutation, increased levels of HSP70 have been observed [62]. A study in patients with chronic heart failure due to DCM revealed a correlation of serum HSP60 levels with disease severity [69]. Since increased levels of HSP27 and HSP70 are associated with a protective effect in models for atrial fibrillation, by maintaining cardiomyocyte function, one can speculate that increased expression of these HSPs might be part of a compensatory protective mechanism in CM [15, 62].

In general, research findings indicate that HSP impairment is detrimental for cardiomyocyte function due to a higher risk of impaired protein folding and aggregate formation. By contrast, HSP activation in CM is considered as a beneficial effect and is most likely a compensatory mechanism of the cell.

### Derailment of the ubiquitin-proteasome system

Derailed UPS function in CM affects the degradation of terminally misfolded proteins. *MYBPC3* mutations often lead to expression of truncated protein, which is not incorporated into the sarcomere because the most C-terminal domain needed for incorporation is missing [64]. Truncated MyBP-C has not been detected in cardiac samples of HCM patients [101]. In addition, very low levels (< 4%) of truncated MyBP-C, which were not incorporated into the sarcomeres, were found in engineered heart tissue made of *MYBPC3* knock-out mouse cardiomyocytes transfected with a truncating *MYBPC3* mutation [110]. Therefore, it is likely that either the mutant mRNA is degraded via nonsense-mediated mRNA decay and/or the truncated MyBP-C forms a substrate for immediate degradation by the UPS or autophagy. Since MyBP-C is highly expressed in cardiomyocytes, high levels of truncated protein may lead to an increased UPS burden and competitive inhibition of the proteasome [84, 85]. In this case, the UPS is overwhelmed by the amount of truncated protein that needs to be degraded. In line with this hypothesis are analyses of myectomy samples from HCM patients with sarcomeric mutations, which show a decrease in proteolytic activity (Table 1) [77, 85]. Decreased processing through the UPS system is also indicated by the increase in overall levels of protein ubiquitination in HCM patients and animal models, which is already detectable at an early postnatal phase prior to any other symptom development [6, 30, 77, 85]. Consistent with the studies in *MYBPC3*-mutant samples, UPS perturbations have also been found in mouse heart tissue with *TNNT2* mutations [30]. Patient samples with a sarcomeric mutation showed higher levels of polyubiquitination and decreased proteolytic activity compared to healthy controls [77].

In addition to overload of the UPS by mutant protein, increased oxidative stress can also impair the function of the proteasome. In this case, the proteasomal dysfunction would not be a direct effect of the mutant protein but a consequence of secondary cellular changes. In CM samples, an increase in oxidation of cytosolic protein content as well as the 19S proteasome, thereby decreasing the overall proteolytic function of the 26S proteasome subunit, has been identified [21, 30, 77].

In addition to the proteasome itself, the expression of ubiquitin ligases can be altered. In an HCM mouse model with mutant *Mybpc3*, the muscle specific E3 ligase Asb2β showed decreased mRNA levels compared to wild-type mice [99]. Since one of its targets is desmin, accumulation of desmin could contribute to the HCM phenotype, as observed for desmin-related CMs.

A large HCM patient cohort and matched healthy controls were screened for genetic variants in all three members of the MuRF family, since mutations in the gene encoding MuRF1 were reported to cause HCM [18]. In this study, a higher prevalence of rare variants of the cardiac-specific E3 ligases MuRF1 and MuRF2 was found in HCM patients [95]. These were associated with earlier disease onset and higher penetrance implying that disturbances of the UPS might act as a disease modifier contributing to HCM.

In contrast to HCM, in DCM, the reported UPS derailments could not yet be linked to sarcomeric mutations. A likely reason for this is that the DCM patient samples did not carry a sarcomeric mutation and/or the underlying disease cause was not known. Tissue analysis from explanted DCM hearts revealed increased expression of both E1 and/or E2 enzymes [47, 108]. Further evidence of increased ubiquitin-conjugating enzyme activity was detected in end-stage DCM. Here, increased levels of MuRF1 and MAFbx were associated with increased UPS degradation activity, which might be the cause of ventricle wall thinning as observed in end-stage DCM patients [9]. In line with the increased ubiquitin-conjugating enzyme levels, increased levels of polyubiquitinated proteins have been detected in DCM samples [11, 47, 72, 108]. This finding is further supported by a 2.3-fold reduced expression of the deubiquitinating enzyme isopeptidase-T in DCM patients [47]. Furthermore, increased proteolytic activity of the 26S proteasome as well as the 20S subunit peptidase activity has been found [9, 11, 72].

In contrast to HSPs, the answer to the question whether UPS activation or inhibition would be beneficial in HCM and DCM is not as straight forward. In an HCM phenotype, proteasome activation might improve the hypertrophic phenotype due to increased mutant protein degradation. However, in DCM, increased proteasome function might augment wall thinning, and therefore, DCM might benefit from proteasome inhibition.

### Unresolved role of the autophagic response in cardiomyopathies

Autophagy is a crucial mechanism in CMs that only fulfills its cytoprotective mechanisms when it is in balance [50]. Moderate activation of autophagy has beneficial effects in CM patients by removing aggregates and supplying the cell with energy.

However, protein degradation due to excessive autophagy has been associated with different types of CM, including HCM, DCM, ischemic CM, and chemotherapy-induced CM [22, 44, 55, 58, 71, 89]. This could lead to loss of myofibrils, as observed in end-stage HCM and DCM patients [40, 70]. In a recent study, the expression of vacuolar protein sorting 34 (Vps34), an important autophagy regulator, was shown to be decreased in the myocardium of HCM patients and deletion of Vsp34 resulted in a HCM-like phenotype in mice. Furthermore, decreased expression of Vsp34 impaired the HSP-autophagy axis, as indicated by αB-crystallin-positive aggregates [44]. HCM patients with mutations in *MYBPC3* or *MYH7* revealed an upregulation of autophagic vacuoles and markers, indicated increased autophagic activity [92]. In a homozygous *Mybpc3*-mutant HCM mouse model, levels of autophagy markers were increased at the protein level implying autophagic activation. However, mRNA levels of these markers were not increased. This rather suggests an accumulation of autophagic proteins due to defective autophagic-lysosomal degradation instead of activation on transcriptional level [85]. In explanted hearts from DCM patients, the imbalance of high ubiquitination rate and insufficient degradation may contribute to autophagic cell death [47]. Vacuolization in CMs has been reported with mutations in *ACTN2*, *MYBPC3*, and *NEBL* [31, 75, 109]. This observation suggests that the accumulation of autophagic vacuoles implies cardiomyocyte stress. However, the interpretation of vacuole accumulation remains unclear, since it could reflect an increase in autophagic activity or an impairment of autophagosome-lysome fusion.

For a correct interpretation of the role of autophagy in CM, autophagic flux in combination with gene and protein expression data have to be studied in the future.

### Environmental stressors influencing the proteostasis network

Besides the above mentioned effects of the sarcomeric gene mutations on the PQC, other environmental stressors, including physiological stress, genetic and epigenetic pathways, and inflammation, can also impair its function [82, 120]. In CM patients with a sarcomeric mutation, these stressors can act as second hit and thereby determine disease severity. Since most of CM patients become symptomatic only in a later stage of their life, the influence of drugs directed at the PQC system as treatment modality for co-morbidities and the age-related decline of the PQC are discussed below.

Several anti-cancer agents block the PQC to cause a lethal proteotoxicity in cancer cells. Anthracyclines, for example, directly impair its function by enhancing proteasomal degradation due to increased expression of E3 ligases and increased proteasome activity as well as inhibition of autophagy in cardiomyocytes [2, 27, 51, 63]. Furthermore, they disturb Ca^2+^ homeostasis, leading to endoplasmic reticulum stress, which derails protein folding [27]. In CM patients, dealing already with a sarcomeric mutation, treatment of another non-cardiac disease can trigger the onset of CM or worsen the clinical outcome. Cardiotoxic side effects of anthracyclines can lead to anthracycline-associated cardiomyopathy (AACM), which presents as LV dysfunction and DCM in adults and RCM in children [53, 61]. Treatment with a low dose of anti-cancer agents induced CM in cancer patients without a history of cardiac disease. Genetic screening of these patients revealed truncating titin variants, which are known as a genetic cause of DCM. These variants may increase the susceptibility for anti-cancer agents-induced CM [52]. In general, patients having a genetic predisposition for DCM are more prone to develop AACM after anthracycline treatment [100, 107]. It can be speculated that the impairment of the PQC due to the anti-cancer treatment is an additional burden to the cardiomyocyte. The clinical cardiac phenotype is caused by insufficient clearance of the mutant protein via UPS and/or autophagy. Therefore, these findings suggest that PQC impairment by anthracyclines can act as catalysts in the development of CM in patients with underlying sarcomeric gene mutation.

Aging represents another cellular stressor leading to toxic mutation effects because of the late disease onset and development of symptoms in inherited CMs. Clinical characteristics, such as wall thickness and diastolic function, worsen with increasing age [57]. This could be related to an age-associated decline in proteostatic function, which is supported by the presence of damaged macromolecules and mitochondria in aged cardiomyocytes [98]. Dysfunctional mitochondria generate high levels of reactive oxygen species, which promote proteotoxic stress and accelerate detrimental effects on the cardiomyocyte [90]. Also, the activity of the 26S proteasome is decreased during aging, which is possibly caused by oxidation of its components [21, 29, 30, 42]. The age-dependent decline in proteasome function increases the burden for the autophagic pathway. However, not only the proteasome, but also the autophagy-lysosomal system declines during aging [81]. As an example, mTOR, a negative regulator of autophagy, was upregulated during aging in a mouse study which indicates decreased autophagic activity [4]. As a result, the activity of the autophagic response might not be sufficient. However, similar to findings related to the UPS, autophagy was enhanced during aging in some animal models, suggesting an increased need for autophagy in aged cells [113]. Further research is warranted to investigate whether the age-related decline of the PQC is causative for CM onset and/or progression.

## Future therapeutic implications

**Fig. 3:**
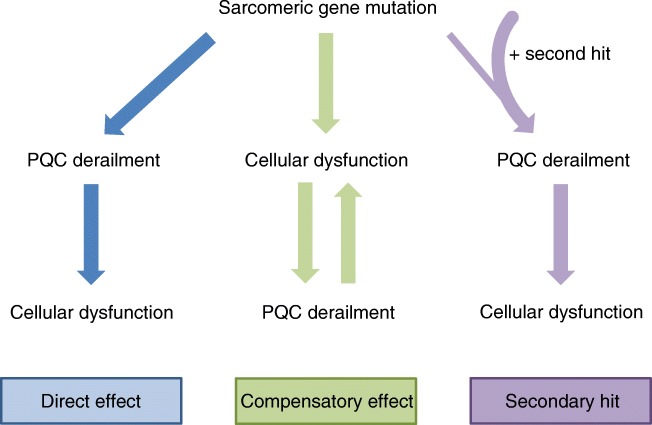
Effects of sarcomeric gene mutations on the protein quality control (PQC) system. Sarcomeric gene mutations can directly derail PQC function leading to cardiomyocyte dysfunction. PQC derailments in cardiomyopathies (CMs) can also be a compensatory mechanism to counteract cardiomyocyte dysfunction caused by the sarcomeric gene mutation. The secondary-hit hypothesis suggests that the PQC of cardiomyocytes carrying a sarcomeric gene mutation is more prone to derail in response to additional cellular stressors, thereby resulting in cardiomyocyte dysfunction

To improve the clinical outcome of CM patients, modulation of PQC components might serve as a novel therapeutic strategy. Figure 3 summarizes the three different ways and illustrates how a sarcomeric gene mutation can lead to PQC derailment. In case of a direct mutation effect on the PQC as well as in combination with secondary hits, targeting of the PQC would be most beneficial and the most direct way to prevent cardiomyocyte dysfunction. In case where the PQC derailment is a consequence of mutation-induced cellular disturbances, it is important to also target the cellular dysfunction to prevent further worsening of the PQC.

As extensively discussed in this review, PQC alterations in CMs are disease- and mutation-specific leading to either increased or reduced function in one or several of its components. Therefore, personalized treatment strategies are required to restore a balanced proteostasis. Potentially, all three PQC components can be therapeutically targeted with the appropriate compounds.

HSP expression can be induced by the drug geranylgeranylacetone. In animal models of desmin-related CM, the induction of HSP expression by geranylgeranylacetone resulted in a beneficial effect on heart function because desmin-aggregate formation was reduced [83]. This example suggests that activation of HSPs might also be beneficial in other types of inherited CMs, since HSPs play a crucial role in coping with the mutant protein.

The derailment of the UPS is context dependent: proteasomal function is decreased in HCM, RCM, and desmin-related CM and increased in DCM [9]. Decreased proteasomal function suggests that the misfolded proteins hamper the UPS by overwhelming it due to permanent degradation of misfolded proteins. As a consequence, the activity of the UPS is reduced. Therefore, UPS activation might be a beneficial therapeutic strategy in HCM and desmin-related CM [9]. In line with this, in HCM patients with a *TNNT2* mutation increased proteasomal activity was correlated with a better clinical outcome [30]. In contrast, over-activation of the UPS indicates a direct response of the UPS to the misfolded proteins to ensure optimal clearance. However, excessive activation of the UPS transforms the initially beneficial effects into a detrimental maladaptation that possibly contributes to loss of myofibrils [9]. Nevertheless, complete proteasome inhibition itself triggered cardiac dysfunction and a CM-like phenotype in healthy pigs [38]. Therefore, it is important to achieve a moderate UPS response in DCM to prevent the detrimental effects of complete proteasome inhibition.

The altered autophagic flux in CMs can be caused on the one hand directly by the misfolded protein itself or on the other hand indirectly by compensating for the impaired functionality of the UPS. To optimize the degradation response, the autophagic activity needs to be pharmacologically titrated into its proteostasis promoting range [114].

## Conclusion

The PQC is crucial for cardiac health and requires the collaboration of all its components to be functional. Key modulators of the PQC are disease- and mutation-specifically altered and derailed in CM. Pharmacological targeting of PQC components represents a novel therapeutic strategy to treat CMs. Since most of the described findings are retrieved from single CM patients or experimental animal models, systematic studies in larger CM patient populations are warranted to untie the knot of disease- and mutation-specific derailments of the PQC.
